# A Mechanochemical Model of Cell Reorientation on Substrates under Cyclic Stretch

**DOI:** 10.1371/journal.pone.0065864

**Published:** 2013-06-06

**Authors:** Jin Qian, Haipei Liu, Yuan Lin, Weiqiu Chen, Huajian Gao

**Affiliations:** 1 Department of Engineering Mechanics, Soft Matter Research Center, Zhejiang University, Hangzhou, Zhejiang, China; 2 Department of Mechanical Engineering, The University of Hong Kong, Hong Kong SAR, China; 3 School of Engineering, Brown University, Providence, Rhode Island, United States of America; University of Birmingham, United Kingdom

## Abstract

We report a theoretical study on the cyclic stretch-induced reorientation of spindle-shaped cells. Specifically, by taking into account the evolution of sub-cellular structures like the contractile stress fibers and adhesive receptor-ligand clusters, we develop a mechanochemical model to describe the dynamics of cell realignment in response to cyclically stretched substrates. Our main hypothesis is that cells tend to orient in the direction where the formation of stress fibers is energetically most favorable. We show that, when subjected to cyclic stretch, the final alignment of cells reflects the competition between the elevated force within stress fibers that accelerates their disassembly and the disruption of cell-substrate adhesion as well, and an effectively increased substrate rigidity that promotes more stable focal adhesions. Our model predictions are consistent with various observations like the substrate rigidity dependent formation of stable adhesions and the stretching frequency, as well as stretching amplitude, dependence of cell realignment. This theory also provides a simple explanation on the regulation of protein Rho in the formation of stretch-induced stress fibers in cells.

## Introduction

**Figure 1:**
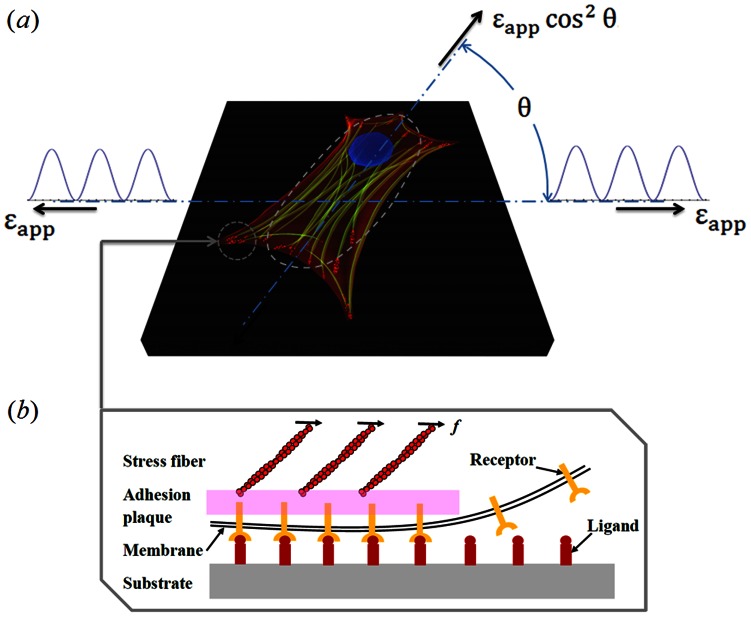
Model description. (a) Illustration of a spindle shaped cell adhered to a substrate subjected to cyclic stretch. The stress fibers (SFs) are largely along the long axis of the cell, anchored at focal adhesions (FAs) near the poles. (b) Schematic drawing of focal adhesions in cell-substrate contact based on specific binding between receptors and complementary ligands. Actin filaments anchor into an adhesion plaque that connects substrate via receptor-ligand bond clusters.

There exists mounting evidence that biological cells have the remarkable ability to sense and react to mechanical cues, although the exact nature of the underlying mechanisms is still largely unknown. For example, it has been shown that strong cell adhesion on extracellular matrix (ECM) cannot be formed when the matrix is softer than a threshold value [1], [2], and consequently, cell locomotion can be guided by rigidity gradient of ECM [3]. Recent observations also demonstrated that, when cultured on a cyclically stretched substrate with oscillating uniaxial strain as depicted in Fig. 1a, cells tend to dynamically reorient themselves and remarkably, different types of cells, including muscle cells [4], [5], fibroblasts [6]–[9], osteoblasts [9]–[11], melanocytes [12] and endothelial cells [13], [14], respond to the imposed stretch in similar fashions.

Given the fact that many organs and tissues, such as heart and artery wall, are subjected to cyclic deformation in physiological conditions, intensive efforts have been spent to investigate why and how cells respond to cyclic stretch, in hopes of shedding light on how processes like angiogenesis take place, as well as finding ways to control or cure various diseases associated with blood vessels and heart in the future. Indeed, several intriguing observations on how cell reorientation is tightly regulated by stretching frequency and amplitude have been reported [4]–[14]. For example, it has been found that, for a cyclic stretch at relatively high frequency (above ∼1 Hz), various cells tend to align nearly perpendicular to the stretching direction when the stretching magnitude is above a threshold value (∼5–6%). However, no apparent cell reorientation was observed when the amplitude of stretching is less than ∼1–2% [4], [7]–[14]. Interestingly, the situation is totally different if the stretch is static or quasi-static (i.e. at very low frequencies), where adhered cells will exhibit distinct modes by aligning themselves either randomly [7] or parallel to the stretching direction [5], [6], [15].

The striking similarity of various cell types responding to cyclically stretched substrates seems to support the hypothesis that cell realignment shares a common physical mechanism. Theoretically, Wang [16] showed that alignment of cells can be explained by assuming that actin filaments have a basal strain energy and any significant deviation from this intrinsic value, induced by applied stretch, leads to filament disassembly. From a different point of view, Chen and Gao [17], [18] considered the problem based on contact mechanics analysis, showing that the adhesion between an elastic cylinder and a stretched substrate exhibits three distinct regimes characterized by two stretch thresholds. Recently, a phenomenological model was proposed by De and Safran [19]–[21] where the central idea is that cells tend to regulate their contractile activities to maintain an optimal stress level in contact with the surrounding matrix. Various theories have also been proposed regarding how cells sense and respond to the stiffness of their surrounding environment, as recently reviewed by Ladoux and Nicolas [22]. For example, the traction dynamics of adhesion clusters formed on substrates with different rigidities has been examined for filopodia [23]. It has been concluded that integrin clustering is robust on stiff matrix but is impaired when the matrix becomes very soft [24]–[26]. Similarly, it has been found that adhesion clusters of receptor-ligand bonds are less stable on more compliant substrates where the effect of stress concentration becomes significant at the adhesion rim [27], [28], and bond rebinding is suppressed due to increased local separation between cell and substrate [29]. Recently, the role of substrate rigidity in the formation and alignment of intracellular actin filaments has also been investigated [30].

Despite all these efforts, several fundamental questions remain unsettled. For example, as evident from the above discussions, the issues of cyclic stretch-induced cell reorientation and how substrate rigidity regulates the behaviors of cell-substrate adhesion were often treated separately in existing studies. Moreover, a limitation of many aforementioned models concerning cell alignment is that no remodeling or evolution of sub-cellular structures has been taken into account. For example, cell reorientation is explained by invoking an effective free-energy, regulated by substrate rigidity, to reflect cells' tendency to achieve “tensional homeostasis” [19]–[21]. It is well-known that strong cell-substrate adhesion is mainly achieved by the formation of stable contact sites, commonly referred to as focal adhesions (FAs), where the so-called ligand-receptor bonds bring cell and substrate together. These bonds are laterally reinforced by a layer of protein complex including vinculin and paxillin [31], often referred to as an adhesion plaque, which interconnects ligand-receptor bonds into clusters and then attaches to the cytoskeleton of cells through stress fibers (SFs) mainly consisting of actin filaments and myosin motors, as shown in Fig. 1b. It is now clear that any reorientation of cells will have to involve the dynamic remodeling of both FAs and SFs. Recently, several researchers [32]–[34] examined this problem by considering the stability of adhesive bond clusters under stretch; however the dynamics of stress fibers was largely unaddressed in their studies. The importance of SFs is evidenced by the role of the protein Rho [35]: inhibition of Rho or its effector proteins such as Rho-kinase and mDia can almost completely block the formation of SFs and, in the present case, a 10% cyclic stretch at 1 Hz was found to cause the reappearance of SFs more parallel to the stretching direction [35]. In contrast, SFs in a normal cell will align nearly perpendicular to the stretching direction [35]. While these observations suggest that the role of stretch in the formation of SFs is actually modulated by Rho, a theoretical explanation is still lacking.

Aiming to address these issues, we have conducted a theoretical study on how and why cells reorient themselves in response to cyclic stretch. Specifically, we develop a coupled mechanochemical model to simulate the temporal response of cell realignment over cyclically stretched substrates, accounting for the dynamic evolutions of both stress fibers and adhesion clusters. The main hypothesis here is that cells tend to orient in the direction where the maximum density of stress fibers, and hence the strongest cell-substrate attachment, can be achieved. We show that, when subjected to a waveform stretch, the final alignment of cells represents a competition between the elevated force within stress fibers that leads to their disassembly, as well as the disruption of cell-substrate adhesion, and the stretch-induced stiffening of the elastomeric substrate which promotes more stable focal adhesions. Our model is capable of explaining a variety of observations like the dependence of cell realignment on stretching frequency, stretching amplitude and Rho regulation.

## Analysis

Consider a spindle-shaped cell adhered to an elastomeric membrane functionally coated with extracellular matrix molecules. The membrane is subjected to a cyclic tensile strain 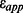 as performed in various experiments [4]–[14]. We use 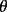 to denote the angle between the stretching direction and the long axis of the cell. In order to achieve successful attachment, the so-called focal adhesions (FAs) must be formed between the cell and membrane at locations near the poles (Fig. 1). These FAs are connected by bundles of actin filaments, called stress fibers (SFs), which allow the cell to exert tractions on the substrate and to probe the mechanical properties of the surrounding environment. When the cell is oriented at an angle 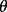 with respect to the stretch direction, the effective stretching strain acting on each SF can be expressed in terms of the applied strain 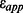 as
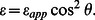
(1)


### Kinetics of FA and SF assemblies

As mentioned earlier, cell realignment involves nucleation and development of both FAs and SFs (Fig. 1b). Here, we describe the formation/disassembly of these sub-cellular structures by monitoring changes in the areal density of ligand-receptor bond 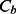 in the adhesion cluster, as well as the density of contracting filament 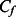 in the stress fiber with time 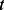, by the first order kinetic equations as
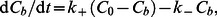
(2a)

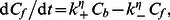
(2b)where 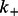 and 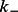 are the association and breaking rates of receptor-ligand bonds, 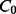 is the maximum bond density that can be possibly achieved in FAs. Similarly, 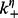 and 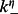 are the association and dissociation rates of contracting filaments. Note that the forward rate of filament growth is assumed to be proportional to the bond density 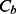 in Eq. (2b), implying that at steady state (if such state exists), the density of contracting filament will be linearly proportional to that of the associated receptor-ligand bonds, a feature that is motivated by the observed correlation between the formation of FAs and SFs [36], [37]. We note that the model by Hsu et al. [14] also considers cell reorientation through dynamic processes of SF assembly and disassembly, as we do in Eq. (2b). The major difference between the two approaches is: Hsu et al. [14] assume a two-dimensional SF network and the growth and shrinkage of the network are directly determined by the strain level in a mathematical expression; while our model focuses on 1D stress fibers in spindle-shaped cells and more importantly, the kinetics of SFs is naturally evolving with substrate stretching through focal adhesions, based on the fact that SFs do not directly link to the substrate.

Treating 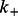, 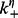 and 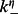 as rate constants, the bond dissociation rate 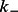 can be expressed as
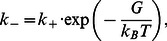
(3)where *G* represents the energy reduction (the more energy reduction, the slower the dissociation process and hence more stable adhesion) associated with the formation of a single ligand-receptor bond, 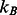 is the Boltzmann constant and 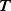 is the ambient temperature in degrees Kelvin. One plausible expression of such energy reduction is
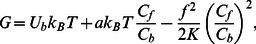
(4)where the first term on the right-hand side corresponds to the energy gained by recruiting one single bond in FAs against membrane fluctuations and steric repulsion of glycocalyx, without the presence of any SFs (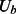 is a dimensionless energy in units of 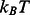). The fact that no stable focal adhesion can be formed in the absence of SFs, i.e. 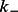 should be greater than 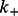 when 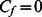, suggests that the value of 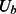 should actually be negative; the second term represents the interaction energy between the ligand-receptor bond and the reinforcing protein whose density is assumed to be proportional to that of the contracting filament (Fig. 1b), with 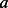 being positive and representing single-pair interaction energy in units of 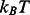; the last term stems from the fact that elastic energy will be stored in the bond-substrate system once a force is present, representing the extra energy needed in forming a single ligand-receptor bond in FA/SF complex. This term takes negative sign because *G* is defined as energy reduction. *K* is the effective spring constant of the bond-substrate system and 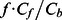 is the averaged load supported by individual bonds, with 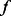 defined as the force generated within each contracting filament.

### Stretch dependent stiffness of the bond-substrate system

**Figure 2:**
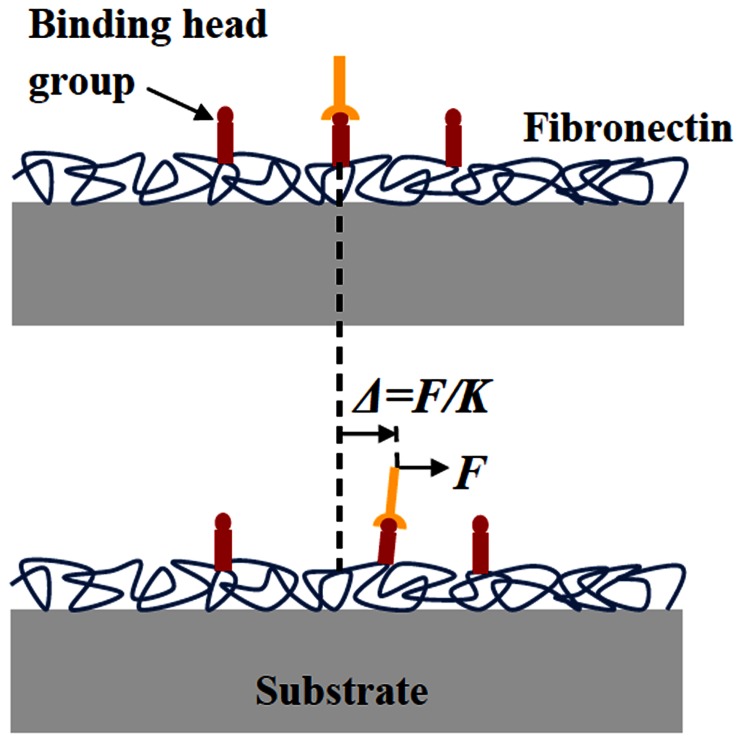
The compliance of the bond-substrate system represented by the effective spring constant *K*. The receptors actually bind to specific head groups of certain adhesion molecules, such as fibronectin, coated on the substrate surface.

Note that the combined stiffness of the bond-substrate system is represented by the effective spring constant *K*. In other words, the bond is expected to displace by a distance 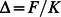 once a force *F* is applied (Fig. 2). A simple scaling argument indicates that *K* can be expressed as
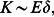
(5)where 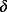 is the diameter of the receptor (∼10 nm according to [38], [39]) and 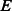 is the combined effective modulus of the substrate and the bond. One important feature about polymeric materials, as some of the soft substrates adopted in experiments [4]–[14], is that their rigidities generally increase with stretching, a phenomenon known as strain stiffening. For example, the moduli of reconstituted actin gels and fibroblasts have all been found to be nearly constant under small strains and increase with the applied strain following a simple power law of index ∼3/2 once the strain level is above a threshold value [40], [41]. Here in our model, 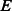 is assumed to depend on the strain 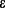 as [40]

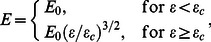
(6)where 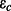 is a critical strain on the order of a few percent, and 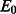 is the modulus value for strains below 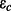. If the parameter 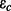 exceeds the maximal stretch the substrate will experience, the material model in Eq. (6) reduces to the case of linear response without any effects of strain stiffening. In the following discussions, we will show that whether substrate stiffening is present or not may lead to distinct cellular responses to very slowly varying stretch, as revealed by several experiments [5]–[7], [15].

### Structural modeling of SFs

**Figure 3:**
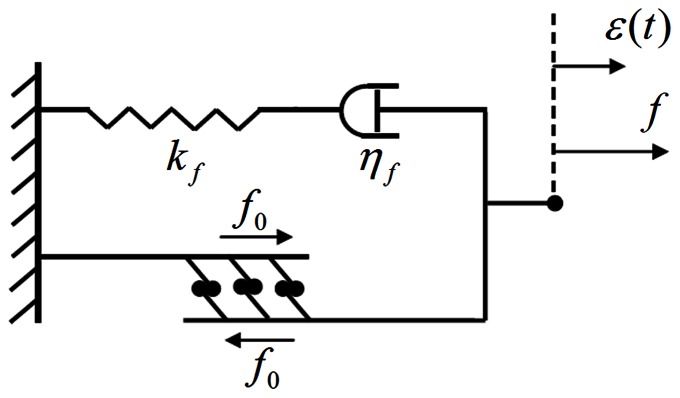
The viscoelastic model of a contracting filament. The structure consisits of a linear spring of stiffness 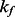, a dashpot of viscous coefficient 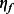 in series, and a parallel module of contraction force 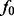.

It is well-known that forces can be generated within SFs due to the activities of associated myosin motor proteins. In addition, forces can also arise from the fact that SFs respond viscoelastically when subjected to external stretching [42], [43]. Based on these understandings, we believe that each contracting filament can be represented by a contracting element from a mechanics point of view, which generates a constant force 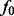 in parallel to an elastic spring and a viscous damper connected in series, as depicted in Fig. 3. It can easily be shown that this description immediately reduces to the well-known Hill's model for muscle contraction [44], [45] if the elastic element, i.e. the spring in Fig. 3, is neglected. By pulling single SFs, experiments [42], [43] have suggested that SFs behave as viscoelastic cables with finite spring constants, so we build the elastic element in Fig. 3 upon Hill's model. Physically, the stiffness 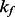 of SFs comes from the ability of actin filaments and crosslinks between filaments in resisting applied forces. Upon application of a waveform loading to the underlying membrane, the cell feels the stretch and the resultant strain in SFs has the form:
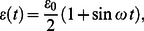
(7)with 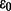 and 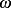 being the stretching amplitude and frequency, respectively. The corresponding strain applied to the membrane, 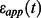, needs to be larger than 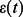 in Eq. (7) in magnitude by a factor of 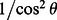 recalling Eq. (1). The assumed model requires that:
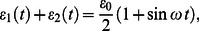
(8a)

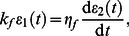
(8b)where 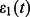 and 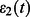 denote the transient deformation in the spring and dashpot, respectively; 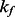 (with dimension of force per strain) and 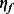 are the elastic and viscous coefficients of the filament. The solution to Eq. (8) leads to the total force generated within each contracting filament:
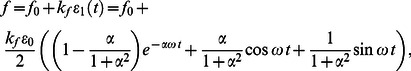
(9)where 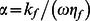. This amount of force is transmitted through adhesion plaque to each receptor-ligand bond, as mentioned in Eq. (4). Note that Eq. (9) is obtained directly from structural considerations of the filament itself, and hence its validity does not depend on how we describe the assembly or disassembly of FAs or SFs. In addition, Eq. (9) also suggests that the force within each filament will always be 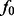 when a static stretch is applied irrespective of the stretching magnitude. We believe that this treatment is not unreasonable since actin filament bundles should be able to relax themselves to accommodate the applied strain by releasing existing or forming new crosslinks given sufficient time.

### Dynamics of cell reorientation and related time scales

For those cells in some orientations that cannot successfully develop long-term stability in FAs and SFs, they will lose contact with the substrate after a short while and then undergo rotational diffusion at random to explore other orientations where formation of new focal adhesions and polymerization of new stress fibers are possible. We assume that 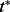 is the characteristic time for nascent focal adhesion, so-called focal complex (FX), to be nucleated in new possible orientations that may or may not lead to mature FAs and associated SFs. The dynamics of cell reorientation is thus described as the loop of orientation search, FX nucleation and FA/SF development, which repeats until certain cell alignment leading to stable FA and SF structures is reached.

In modeling rotational diffusion at random of whole-cell, the mean-square angular deviation in elapsed time 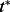 is 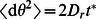 according to [46], which has exactly the same form as the classical one dimensional model of translational Brownian motion. The difference here is that 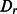 is the rotational diffusion coefficient in units of radian^2^/s. In an alternative description of equivalence, a cell will hop by an angle
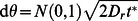
(10)about the cell center in time 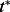 between losing adhesion at old orientation and nucleating adhesion at new orientation. Here 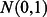 is a random number following normal distribution with zero mean and unit variance.

## Results and Discussion

### Normalization of governing equations and estimate of parameters

We proceed by normalizing the physical parameters as 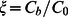, 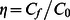, 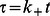, 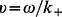, 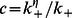, 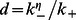. Hence, Eq. (2) becomes
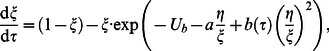
(11a)

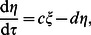
(11b)where 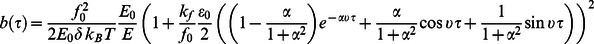 with 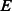 explicitly given by Eq. (6).

The time needed for the formation of single receptor-ligand bonds, characterized by the bond association rate 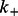, is of the order of 0.01–1 second [47], [48], which serves as a reference time scale in the model. Here we choose 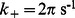. The formation of stress fibers should be slower than the association of bonds as it generally involves more complicated processes of actin polymerization as well as the assembly of associated myosin molecules, which plays an essential role in the dynamics of cytoskeleton [49], [50]. Furthermore, it is not unreasonable to believe that the densities of bonds and contracting filaments are comparable, hence the dimensionless parameters *c* and *d* are all estimated to be of the order of 0.1, following the observations that the time for the disassembly/elongation of SFs is of the order of minutes [51], [52]. Cytoskeletal fluidization in response to mechanical force was investigated in the series of work by Fredberg group [53]–[55]. In their experiments, smooth muscle cells were stretched via biaxial or uniaxial deformation applied to the gel substrates. In both cases, cytoskeleton fluidized at a time scale of ∼4 seconds immediately after a transient strain of ∼10% to the substrate [53], [54], indicating a prompt process of stress fiber disassembly. Fredberg's experiments confirmed that the FA tractions were markedly ablated due to the applied strain. With vanishing 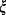 (FA density) in our model, Eq. (11) reduces to 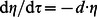 with 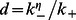, where 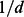 serves as the characteristic time scale of SF disassembly. In our calculation, 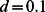 corresponds to a time scale of ∼1.6 seconds according to our normalization scheme, on the same order of magnitude as experimentally revealed.

The energy reduction by forming a single bond is around 5–10 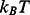 [56], [57], and similarly, it can be expected that the interaction energy between a bond and a reinforcing protein is of the order of a few 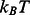 as well. Hence, we proceed by choosing *a*, dimensionless energy in units of 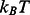 referring to Eq. (4), to be 3.5. In addition, we notice that factors like thermally induced membrane fluctuations and possible presence of flexible molecules, such as glycocalyx, tend to disrupt any adhesion between the cell and substrate. As discussed above, 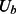 in Eq. (4) should be negative and our calculations are conducted with 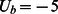. It has been found that the traction level acting on individual FAs is surprisingly uniform across cell types [37], [58]–[63], and we take the value 3 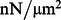 in our calculations. In addition, the bond spacing within FAs is assumed to be around 20 nm representing a bond density of ∼2500 per 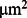. As such, 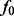 is estimated to be of the order of 1 pN here. The spring constant of SFs has been experimentally measured to be around 45 nN per unit strain [42], so 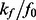 is estimated to be ∼20 given the fact that the characteristic diameter of FA is around 1 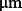 [37]. The typical relaxation time of stress fibers is of the order of a few seconds [43], so we expect 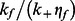 to be around 0.5. The effective bond-substrate modulus 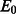 is hard to evaluate; however, it is safe to believe that its value is between ∼5 kPa, the modulus of soft polyacrylamide substrates [2], and ∼500 kPa, the modulus of polydimethylsiloxane (PDMS) [64]. As such, the value of 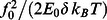 is estimated to fall into the range of 0.03 to 3. In the present scheme of normalization, the dimensionless angular hopping time 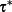, i.e. 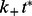, is chosen to be 0.2, and a cell will rotate by 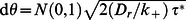 before nascent focal complex is nucleated at new orientations. Due to the separation in length/time scale, whole-cell is expected to reorient much more slowly than the processes of FA evolution and SF remodeling that happen at molecular scale, so that 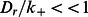. The following calculations are all based on these representative values of involved parameters unless specified otherwise, as gathered in Table 1.

**Table 1:** Estimated values of the parameters in the model.

Notation	Meaning	Value
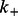	Association rate of ligand-receptor bonds	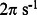
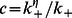	Normalized association rate of stress fibers	0.2 (0.1 for Rho-inhibited cells)
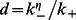	Normalized dissociation rate of stress fibers	0.1
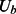	Normalized energy reduction by forming a single ligand-receptor bond	−5
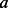	Normalized interaction energy between a bond and a reinforcing protein	3.5
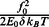	Dimensionless parameter representing the substrate compliance	0.6
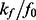	Normalized effective spring constant of actin filaments	15
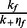	Dimensionless ratio between the elastic and the viscous coefficients of actin filaments	0.5
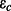	Critical strain for strain hardening to take place in substrate	4% ( 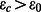 for linear substrates)
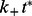	Characteristic time scale associated with nucleation of nascent FA (normalized)	0.2
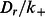	Normalized rotational diffusion coefficient	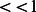
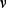	Poisson's ratio of substrates	0, 0.5

### Formation of FAs and SFs regulated by substrate rigidity

**Figure 4:**
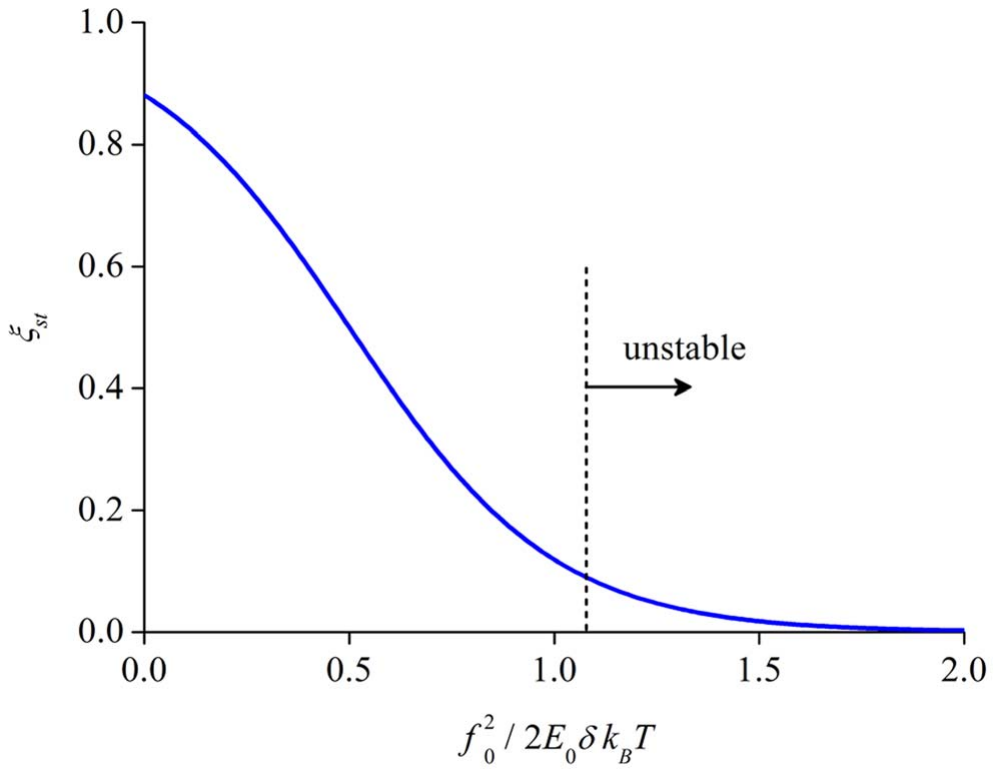
Steady state bond density as a function of substrate rigidity.

Let's first neglect any externally applied stretch, correspondingly 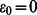, and investigate how cells sense and respond to substrate stiffness on their own contractility (described by 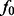) through the competing process between formation and disruption of FAs and SFs in our modeling framework. From Eq. (11), it is clearly seen that the steady state solution is
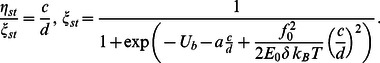
(12)Plotted in Fig. 4 is 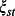 as a function of 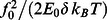 for estimated values of 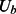, *a* and 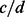 in Table 1. Obviously, 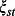 and hence 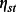 decrease exponentially as the substrate, or more precisely the bond-substrate system, becomes more compliant, in qualitative agreement with Saez et al. [65] showing that the traction forces developed by cells increase as the substrate becomes more rigid. Physically, this can be understood by realizing that the strain energy stored, or equivalently the amount of energy cells need to invest, in the bond on a softer substrate is higher than that on a more rigid, which makes the formation of bonds on softer substrates more energy-consuming.

**Figure 5:**
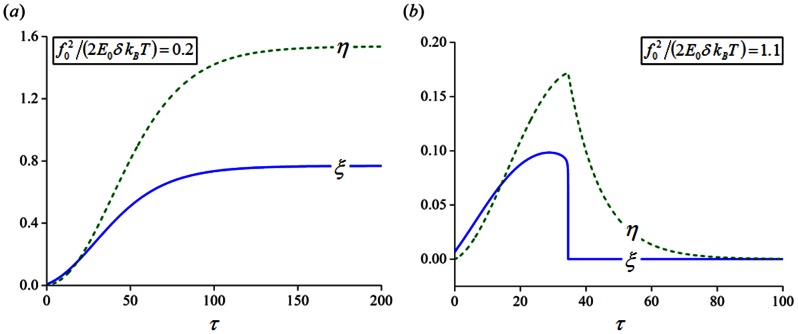
Evolutions of the bond and filament densities. (a) A relatively stiff substrate of 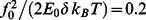. (b) A relatively soft substrate of 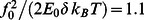.

By choosing initial conditions as 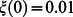 and 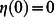, the dynamic evolutions of 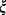 and 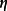 when 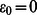 are shown in Figs. 5a and 5b, for 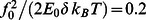 and 1.1 respectively. Related to biological aspects, the nonzero initial value of 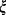 is considered to arise from nascent nucleation of focal complex that may or may not lead to mature FAs and associated SFs, as discussed in previous sections. Clearly, the steady state solution given by Eq. (12) can indeed be achieved when 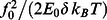 is relatively small, or equivalently when the substrate is more rigid. However, as the substrate becomes more compliant, the steady state solution becomes unstable when 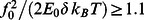, that is any tiny fluctuations will cause the bond, as well as the contracting filament, density to suddenly drop to zero, referring to Fig. 5b. Notice that 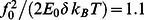 roughly corresponds to a substrate with rigidity around ∼15 kPa. Interestingly, it has been reported that both normal rat kidney (NRK) epithelial and 3T3 fibroblastic cells cannot form stable FAs on substrates with modulus less than ∼10 kPa, while these cells can firmly attach to substrates with stiffness of ∼60 kPa and above [2], in broad agreement with the theoretical predictions here. We have to point out that similar conclusion has also been obtained recently by Paszek and co-workers [26], who showed that clustering of integrins, a key step in the formation of stable adhesion clusters, is greatly impaired when the substrate is softer than a threshold value.

### Stretch frequency and amplitude dependent reorientation of cells

**Figure 6:**
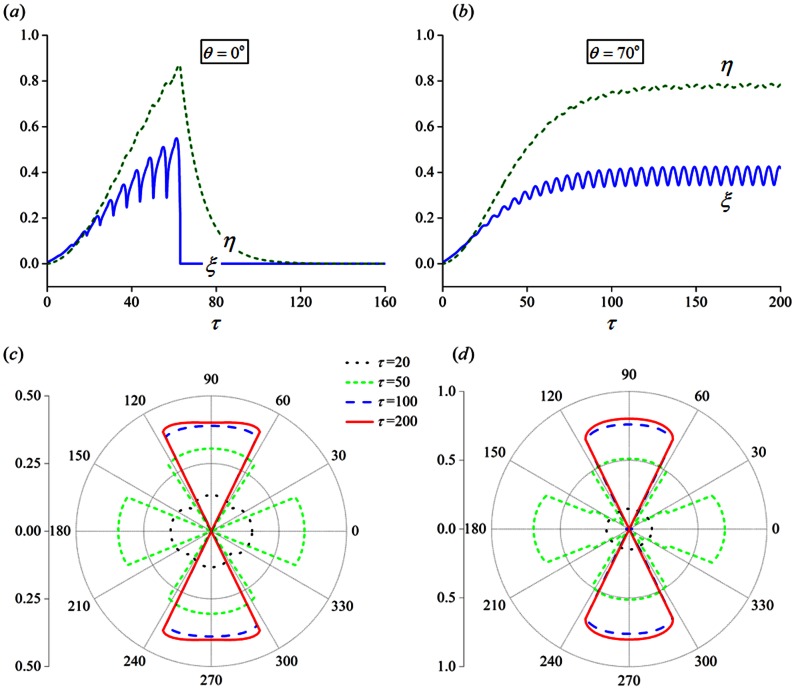
Evolutions of the bond and filament densities corresponding to different cell orientations. (a) 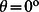 (parallel to stretch direction). (b) 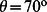 (nearly perpendicular to stretch direction). (c, d) Evolution snapshots of (c) the bond and (d) filament densities, represented by the radial distance from the origin, as a function of cell orientations (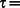
20, 50, 100 and 200, respectively).

When subjected to a cyclic stretch 10% in amplitude and 1 Hz in frequency, or 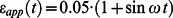 with 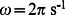, the evolutions of 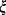 and 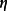 corresponding to cell orientations of 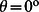 and 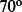 are shown in Figs. 6a and 6b, respectively, when 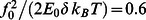. Clearly, in spite of the oscillations induced by the cyclic straining, the values of 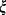 and 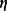 are stably maintained at constant levels when cells are aligned away from the stretching direction at 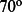 (Fig. 6b). However, the situation becomes unstable when cells are oriented parallel to the stretch axis (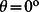), where both 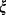 and 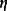 abruptly drop to negligible levels after a short while (Fig. 6a). Basically, for a 10% stretch at 1 Hz, long-term stability in FAs and SFs is possible only within the angle region close to the direction perpendicular to stretch (Figs. 6c and 6d), which explains why many cell types tend to reorient themselves away from the stretching direction, as reported in various studies [4], [7]–[14].

**Figure 7:**
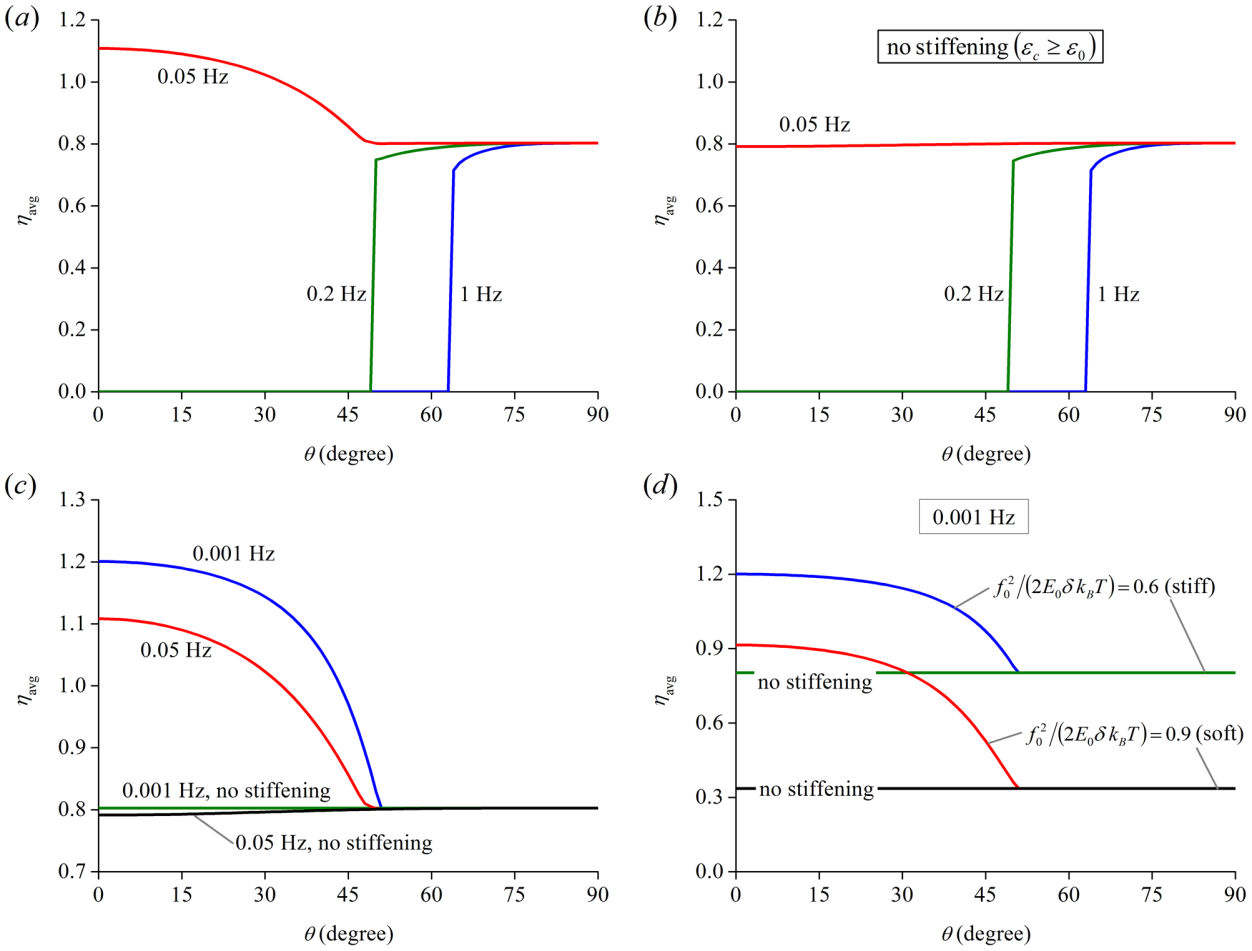
Long-time average filament density 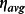 as a function of the cell orientation angle 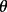 under a 10% stretch at different frequencies. (a) 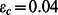. (b) 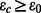 where strain stiffening is not present as the substrate is stretched. (c, d) Effects of (c) strain stiffening and (d) substrate rigidity on 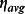 for low frequencies (0.05 Hz and 0.001 Hz, respectively).

If we denote 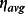 as the long-time average value of 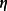, Fig. 7a plots 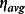 as a function of orientation angle 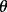, and also shows how 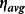 varies with respect to 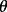 when the stretching frequency is reduced from 1 Hz to 0.2 and 0.05 Hz. Obviously, stable SFs can be formed in all orientations when the stretching frequency is low enough, say below 0.05 Hz. Furthermore, under such circumstance, the maximum density of SFs is achieved when cells are aligned parallel to the stretch axis (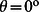), in direct contrast to cases where the stretching frequency is relatively high. Physically, this can be understood by realizing that contracting filaments have enough time to relax the imposed strain when 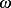 is small and hence the force within them remains more or less unchanged at constant level 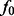. As such, the effect of stretch-induced hardening of substrate will outweigh that introduced by the elevation in filament force, eventually causing more FA bonds as well as more SFs to form. It is conceivable that cells prefer to orient in the direction where the densities of both SFs and FAs are maximized and consequently, the strongest cell-substrate attachment is achieved. If we accept this hypothesis, then Fig. 7a suggests that cells are more likely to align themselves along the stretching direction when the stretch is static or quasi-static, a prediction consistent with experimental observations [5], [6].

**Figure 8:**
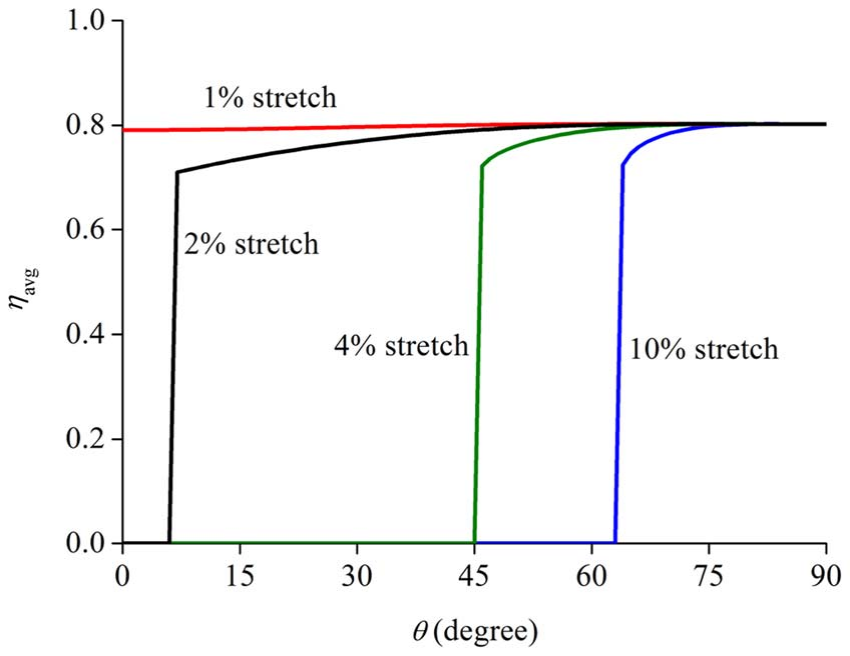
Long-time average filament density 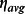 as a function of the cell orientation angle 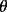 under different values of stretching amplitude (stretch frequency: 1 Hz).

Alternatively, when the stretching frequency is fixed at 1 Hz, Fig. 8 shows the variation of 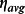 as functions of cell orientation 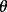 under different stretching amplitudes. An immediate observation is that 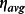 becomes insensitive to 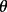 when the stretching amplitude is small (∼1%), suggesting that cells do not have a preferable orientation in this case. Interestingly, it has been reported that cells indeed do not respond to stretches with amplitude less than ∼1–2% [4], [7]–[14].

We have also carried out calculations for the case of substrates with linear response, i.e. constant substrate modulus irrespective of stretch amplitude, which are compared to the aforementioned results where strain stiffening is present (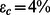). The observations are the following: i) Adopting linear response in substrate does not influence stretching amplitude dependence of cell realignment (Fig. 8), which is obvious because stiffening effects are not present even for non-linear substrates when 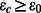. ii) For stretching frequency dependence of cell realignment (Fig. 7), the two cases, with and without strain stiffening for substrate materials, do not differ for relatively high frequency (>0.2 Hz), but interestingly, exhibit two distinct modes for low frequencies, referring to Figs. 7a and 7b. Both random cell orientation and preferred alignment in the stretch direction were observed in experiments [7], [15]. There are discussions suspecting that the difference in substrate properties of the two studies, stiff PDMS [7] versus soft collagen lattice [15], may cause the distinct modes of cell reorientation. Our results show that, irrespective of the base-line rigidity 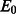, strain hardening in the substrate leads to cell alignment along the stretching direction when low frequency stretch is applied. In comparison, random cell orientation is expected if the response of substrate is linear (Figs. 7c and 7d). Hence, according to our model, the different orientation patterns observed in [7] and [15] might be due to that collagen will undergo strain stiffening while the response of PDMS remains more or less linear. Interestingly, the original paper by Brown et al. [15] used collagen gels of 2.28 mg/ml native rate tail type I collagen without doing mechanical testing on it; while a later study by Storm et al. [66] showed that rat tail type I collagen of 2 mg/ml exhibited significant strain stiffening at <10% stain and 10 radian per second (∼1.6 Hz). We should point out that what Storm et al. reported was the shear modulus of collagen gels. Given the explicit relation between shear modulus and Young's modulus, we expect the similar non-linear behavior for collagen gels under axial stretching.

### Reorientation of multiple cells with initially random alignment

Jungbauer et al. conducted the first detailed experimental investigation of the temporal reorientation of multiple cells in response to cyclic substrate stretch of various amplitude and frequency [7], where two different types of fibroblasts were periodically stretched via 1–15% strain at 0.0001–20 Hz in elastomeric substrates. The following observations have been made from their work [7]:

- The characteristic time for the dynamic reorientation is frequency-dependent and is within a range from 1 to 5 hours, indicated by real-time track of the order parameter;
- For dependence of stretching amplitude, the characteristic time of cell reorientation increases linearly with reducing amplitude, and no apparent cell reorientation is observed when the amplitude of stretching is reduced to below ∼1–2%;
- The orientation change of multiple cells occurs faster at higher frequencies, and a threshold frequency, ∼0.01 Hz for rat embryonic fibroblasts and ∼0.1 Hz for human dermal fibroblasts, is found below which no significant cell reorientation occurs;
- More interestingly, a biphasic relation is found between the characteristic time of cell reorientation and stretching frequency for both cell types, with a generic threshold frequency ∼1 Hz separating the two phases.

**Figure 9:**
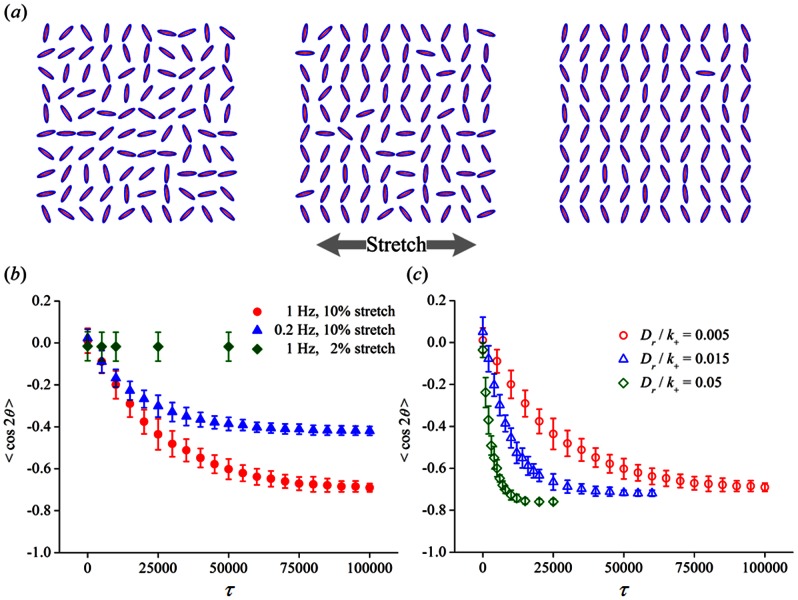
Reorientation of multiple cells on cyclically stretched substrates. (a) Alignment of 100 individual cells adhered to a substrate which is subjected to a 10% stretch at 1 Hz. The stretch is applied along the horizontal direction and from left to right, the order parameters corresponding to particular time points are: 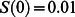, 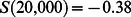 and 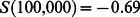. (b, c) Dynamic evolution of the order parameter 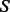 representing instantaneous cell orientation of 100 cells on the cyclically stretched substrate at different values of straining frequency, amplitude and rotational diffusivity. Each error bar reflects the standard deviation (SD) of 10 independent sets of simulation.

To quantitatively compare our model to the experimental results, the order parameter
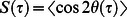
(13)is calculated for instantaneous cell alignment of 100 independent cells per simulation, as have been monitored in the experiments [7]. Theoretically, the limiting case 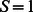 indicates that all the cells are orientated parallel, 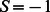 indicates a perpendicular alignment with respect to the stretch axis, and 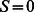 corresponds to a perfectly random orientation of cells. In our simulation, the cells are initially orientated at random, corresponding to 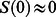 (left portion of Fig. 9a). Starting the stretch with 10% in amplitude and 1 Hz in frequency at the onset of simulation, the cells realign themselves more and more perpendicular to the stretching direction as time elapses, as reflected by the snapshots in Fig. 9a and the order parameter 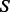 changing from an initial value near zero to a steady state level around −0.7 in Fig. 9b. By examining the frequency and amplitude dependence of the temporal response of cells, we find

- The characteristic time for cell reorientation to occur is largely determined by the parameter 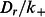, the slowest one among all the involved time scales in our modeling framework, as indicated in Fig. 9c. Given the reference time scale 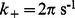 we adopt, the observable realignment of cells occurs in time of hours, which is comparable to the experimental observations that noticeable cell reorientation was observed to occur from 1 to 5 hours for fibroblasts [7], within 24 hours for osteoblasts [9] and from 1 to 2 hours for endothelial cells [14];
- Cell reorientation is almost unnoticeable when the amplitude of stretching is reduced to below 2%, referring to Fig. 9b;
- Cell reorientation is found to occur faster but the steady state value of the order parameter is smaller in magnitude at a lower stretching frequency of 0.2 Hz (Fig. 9b), consistent with observations for fibroblasts [7];
- Fitted Fig. 9b by the same exponential expression as the one used by Jungbauer et al. [7], we obtain that the characteristic time of *S* decay is 26,000 for 1.0 Hz and 19,000 for 0.2 Hz, differing by ∼40% but not as significant as the observation of the biphasic dependence of time required to achieve steady state as a function of frequency [7]. We believe that the problem may be caused by some hidden temporal processes with extra time scales, whose nature is unclear to us at current stage, or it can be due to the possibility that FA/SF kinetics is not fully captured by the first-order rate equations and higher-order modeling is needed.

### Effects from Poisson's ratio of the substrates

**Figure 10:**
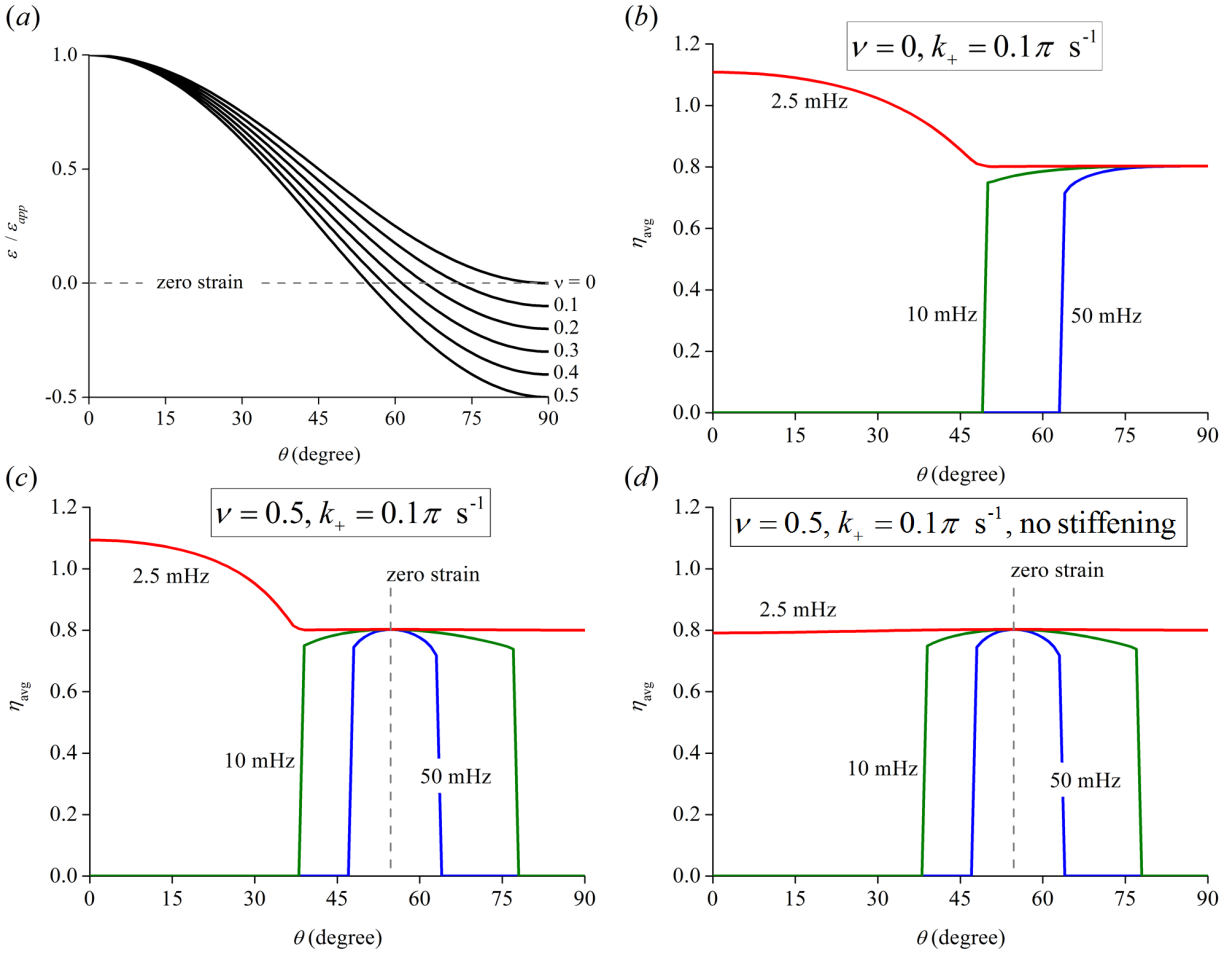
Effects of Poisson's ratio on cell reorientation. (a) The effective stretching strain acting on each SF as a function of cell orientation angle 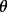, influenced by Poisson's ratio 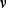 of substrate materials. (b, c, d) Long-time average filament density 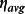 as a function of the cell orientation angle 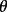 for slow kinetic process (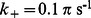) and (b) 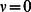, (c) 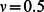 and (d) 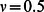 without strain stiffening effects, respectively.

There have been studies focusing on the effects of transverse deformation in substrates on cell reorientation when the substrates are stretched in the axial direction [67]–[69], called the Poisson effect. For elastic and isotropic materials, Poisson effect is quantitatively measured by Poisson's ratio 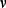, which is a material constant defined as the negative ratio of the strain in the transverse direction perpendicular to the applied strain. 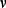 falls into the range between 0 and 1/2 for most materials. When Poisson effect of the substrate materials is significant, the effective stretching strain acting on each SF in Eq. (1) should be extended to
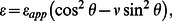
(14)referring to Fig. 10a. In other words, the results in previous sections are valid under the assumption that 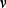 is close to zero. The study by Faust et al. [67] differs from others in two aspects: a) cyclic stretch at tens of mHz was found to cause cells to reorient away from the stretching direction, one order of magnitude lower than most other studies and implying a much slower temporal process of FAs and SFs; b) more interesting, while substrates with Poisson's ratio 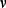 close to 1/2 were used, cells were observed to more align in the direction of zero effective strain, instead of the direction perpendicular to the stretching direction. We perform calculations by taking 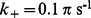 for slow kinetics and 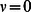 and 0.5, respectively, for zero and strong Poisson effect in substrate deformation (Figs. 10b and 10c), with the results showing that cells prefer to align in the zero strain direction and not in the direction perpendicular to the applied stretch from FA/SF stability point of view, consistent with Faust et al. [67]. Furthermore, the preferred cell alignment in the zero strain direction doesn't rely on strain stiffening in the substrates, as confirmed in the case where 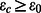 and stiffening effects are not present (Fig. 10d).

### Possible connections to the role of Rho in SF formation

**Figure 11:**
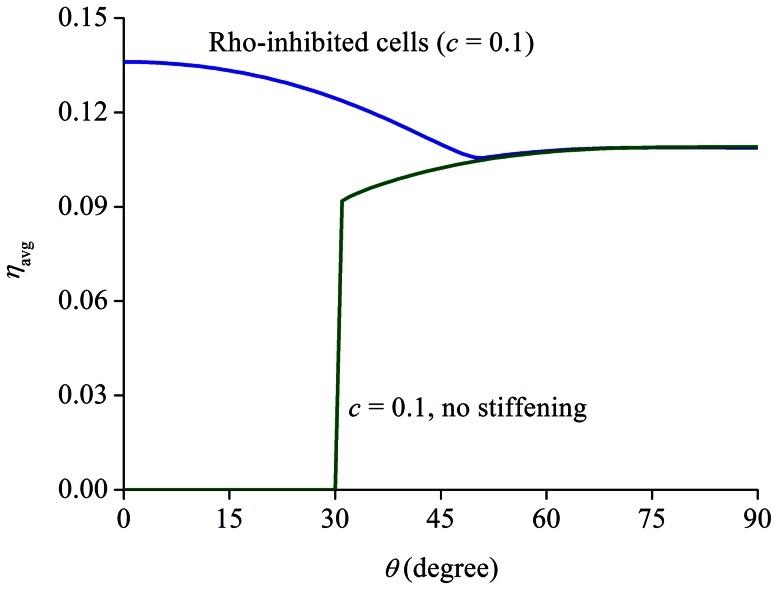
Long-time average filament density 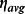
 **in “Rho-inhibited” cells (** 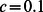 **) as a function of the orientation angle** 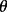 **under a 10% stretch at 1 Hz.** The results clearly show the difference between the cases with and without strain stiffening effects.

As pointed out earlier, it has been shown that inhibition of Rho or its effector proteins almost completely blocks the formation of SFs. However, a 10% cyclic stretch at 1 Hz causes the reappearance of SFs more along the stretching direction, having much lower SF density compared to that in normal cells [35]. Within the framework of our model, let's assume that the effect of Rho inhibition can be represented by a reduction in the value of parameter *c* that appears in Eq. (11), which effectively decreases the steady state density of contracting filaments. We proceed by choosing 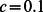 for Rho-inhibited cells, comparing to 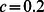 for normal ones. When subjected to a 10% stretch at 1 Hz, 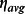 as a function of 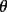 is shown in Fig. 11. Several important observations are: (1) the filament density here is almost 6–9 times lower than that in normal cells, refer to Fig. 7 or 8; (2) more interestingly, the maximum SF density is achieved when these “Rho-inhibited” cells align parallel to the stretching direction. Actually the SF density increases by almost 20%, from ∼0.11 to ∼0.135, when a cell flips its orientation from perpendicular to parallel to the stretch axis, where no effective stretch is acting on the cell. Hence, our model provides a simple qualitative explanation on why a cyclic stretch can lead to the reappearance of SFs more along the stretching direction in Rho-inhibited cells. Moreover, our results show that whether strain stiffening is present or not also leads to two modes: cells will tend to align parallel or perpendicular to the stretch direction (Fig. 11). The fact that SFs are more parallel to the stretch direction seemingly suggests that the substrate used in the experiment [35], silicone rubbers, is non-linear in elastic response.

We must point out that the treatment adopted here to represent the effect of Rho inhibition is extremely simple given the complexity of cell biology. Essentially, what we have demonstrated is that by adjusting one single parameter according to well-known unique features of Rho-inhibited cells in comparison to normal ones, our model is capable of explaining how and why these cells respond to cyclic stretch in certain manners, as observed in experiments [35].

## Conclusions

In this paper, we have reported a detailed quantitative examination on how and why various cell types reorient in response to cyclically stretched substrates. Specifically, by taking into account the dynamic evolutions of sub-cellular structures such as stress fibers and receptor-ligand bond clusters, a mechanochemical modeling framework has been developed to predict the preferred alignment of cells under stretch. Our main hypothesis is that cells tend to orient in the direction where the maximum density of stress fibers, and hence the strongest cell-substrate attachment, can be achieved. We show that, for a cell-substrate system subjected to cyclic stretch, the final alignment of cells represents the competitive coupling between stress fiber assembly/disassembly, focal adhesion growth/disruption, substrate stiffening and whole-cell rotation. Our model is capable of explaining a broad range of observations like the absence of stable FAs or SFs on sufficiently soft substrates as well as the stretching frequency and amplitude dependent realignment of cells. In addition, this theory provides a possible explanation on the stretch-induced reappearance of SFs in Rho-inhibited cells.

Several important aspects of the problem have been neglected in the present study, which certainly warrant future investigation. For one thing, the role of myosin motors is represented by the generation of a constant force in the contracting filaments in contact with SFs' viscoelastic response, which certainly might be an oversimplification of the real system of filament-myosin architecture. A more realistic formulation should explicitly take into account the activities of filament-myosin assembly during the time-varying stretch as well as its implications on the evolution of SFs. For another, one of our hypotheses is that cells tend to orient in the direction where the maximum densities of ligand-receptor bond and actin contracting filament are achieved. In other words, our results reflect this preferred criterion of final cell alignment but have done little to address the question of how cells actually conform themselves to achieve so, which is certainly an important and interesting task to pursue in the future. In addition, the diffusion and possible self-aggregation of adhesion molecules in FAs have been neglected in our analysis. However, it is commonly believed that the recruiting and clustering processes of these proteins, such as integrins, are important for the formation of stable focal adhesions.

Despite all the limitations mentioned above, we feel that our simple model successfully establishes a connection between the evolutions of sub-cellular structures, like adhesion clusters and stress fibers, and various mechanical factors such as substrate rigidity, SF viscoelasticity and imposed stretch. The validity of the key assumptions made in our formulation, like the viscoelastic response of SFs and the strain stiffening of polymeric substrates, has been well confirmed by existing studies. The fact that the predictions from our model compare favorably to a variety of experimental observations suggests that the main physics of the problem under investigation may have been captured by this formulation, which we believe can serve as a theoretical framework to motivate future studies on cell reorientation, where further verification of the main mechanisms and more realistic features can be added.
